# Genome-Wide Identification of CFEM Proteins in *Sclerotinia sclerotiorum* Reveals Effector Candidates with Cell Death Suppression Activity

**DOI:** 10.3390/plants15060957

**Published:** 2026-03-20

**Authors:** Xihong Li, Yuting Wu, Linxuan Liu, Shuang Liu, Dan Zhang, Xianfeng Yi, Lele Wang, Shan Liu, Rongchao Jia, Jinpeng Shi, Stefan Olsson, Congcong Lu, Airong Wang, Ya Li

**Affiliations:** 1 State Key Laboratory of Ecological Pest Control for Fujian and Taiwan Crops, College of Plant Protection, Fujian Agriculture and Forestry University, Fuzhou 350002, China; 15286807308@163.com (X.L.); yutingwwu@126.com (Y.W.); liulinxuan817@163.com (L.L.); ls17530377141@126.com (S.L.); zd@fafu.edu.cn (D.Z.); yxf@fafu.edu.cn (X.Y.); wll13592549772@163.com (L.W.); lsls@fafu.edu.cn (S.L.); jiarongchao1@163.com (R.J.); 18336885220@163.com (J.S.); stefan.olsson.kvl@gmail.com (S.O.); 2 Key Laboratory of Biopesticide and Chemical Biology (Ministry of Education), College of Plant Protection, Fujian Agriculture and Forestry University, Fuzhou 350002, China; 3 Fujian Universities Key Laboratory for Plant-Microbe Interaction, College of Life Science, Fujian Agriculture and Forestry University, Fuzhou 350002, China

**Keywords:** *Sclerotinia sclerotiorum*, CFEM domain, bioinformatics analysis, effector prediction, expression profiling

## Abstract

The CFEM (Common in Fungal Extracellular Membrane) domain defines a family of cysteine-rich proteins unique to fungi, playing pivotal roles in host–pathogen interactions. However, the repertoire and functions of CFEM proteins in the broad-host-range necrotrophic pathogen *Sclerotinia sclerotiorum* remain largely unexplored. Through genome-wide bioinformatic analysis, we identified 13 CFEM-containing proteins (SsCFEM1–13) in *S. sclerotiorum*. Characterization revealed substantial diversity in their physicochemical properties, domain architecture, and predicted subcellular localization. Ten proteins possess a secretion signal, with six predicted to be GPI-anchored and three classified as high-confidence effectors. Members lacking transmembrane domains were predicted to adopt the conserved CFEM “helical-basket” fold. Phylogenetic analysis grouped SsCFEMs into two distinct clades and indicated a complex evolutionary history involving both conserved ancestry and lineage-specific expansion. Transcriptomic profiling showed that most genes were upregulated during early infection of various host plants, with *SsCFEM8* exhibiting particularly strong and consistent induction. Crucially, transient expression assays in *Nicotiana benthamiana* revealed that several SsCFEM proteins, notably SsCFEM4 and SsCFEM9, function as cell death suppressors, validating their predicted effector roles and identifying key virulence candidates. This study provides the first comprehensive catalog and functional prediction of the CFEM protein family in *S. sclerotiorum*, establishing a foundation for future mechanistic studies on their roles in the pathogenesis of this devastating fungal pathogen.

## 1. Introduction

The CFEM (Common in Fungal Extracellular Membrane) domain is a fungi-specific cysteine-rich extracellular domain characterized by the presence of eight conserved cysteine residues that form a specific disulfide bond network, stabilizing its three-dimensional structure [1]. Initially identified through the analysis of over 25 fungal protein sequences, its structural features are distinct from other known cysteine-rich domains, suggesting a unique role in fungal biology [1]. Subsequent systematic phylogenomic analyses confirmed that the CFEM domain is indeed unique to fungi, with a single origin dating back to the most recent common ancestor of *Ascomycota* and *Basidiomycota*, rather than multiple independent origins [2]. The length and architecture of this domain are relatively conserved, but its copy number varies significantly among different fungal species. Notably, pathogenic fungi generally possess a larger number of CFEM domains, implying a potentially important role in host–pathogen interactions [2].

In human pathogenic fungi, CFEM proteins have been extensively characterized, especially in *Candida albicans*, where they are crucial for iron acquisition—a key virulence determinant. The GPI-anchored proteins Rbt5 and Pga7 bind heme and facilitate its utilization from hemoglobin, forming a relay network that delivers heme to the endocytic machinery [3,4]. Another CFEM protein, Csa2, also supports heme-iron uptake, particularly during hyphal growth [5]. Proteomic studies confirm that several CFEM proteins are upregulated under iron limitation, and their domains directly bind heme, underscoring their central role in iron metabolism [6]. This heme-acquisition system functionally cooperates with ferric reductase-related proteins (Frp1/Frp2), suggesting an evolutionary adaptation from ancestral reductases [7]. Beyond iron uptake, CFEM proteins in *C. albicans* contribute to biofilm integrity, cell surface architecture, and adhesion, with mutants showing increased sensitivity to wall stressors and altered hydrophobicity [8,9]. Their expression is induced by hypoxia and sterol inhibitors, regulated by transcription factors Upc2 and Bcr1 [10]. Functional conservation and divergence are observed in other pathogenic yeasts: While *C. parapsilosis* CFEM proteins retain roles in iron acquisition, their involvement in biofilm formation differs [11], and in *C. glabrata*, the CFEM protein CgCcw14 is vital for iron homeostasis, host cell adherence, and virulence [12].

In plant pathogenic fungi, CFEM proteins are established key virulence factors with multifunctional roles in host recognition, infection structure development, immune suppression, and nutrient acquisition. In necrotrophic pathogens such as *Botrytis cinerea*, CFEM proteins like BcCFEM1 are upregulated during early infection and contribute to pathogenicity, conidiation, and stress tolerance [13]. A non-GPCR membrane-bound CFEM protein in the same fungus also influences lesion progression and conidial germination [14]. Comparative secretome analyses further suggest that CFEM-containing effectors may differ among necrotrophs, reflecting pathogen-specific adaptation [15]. In biotrophic/hemibiotrophic pathogens, CFEM proteins exhibit even greater functional diversity. In *Magnaporthe oryzae*, CFEM-containing GPCRs such as Pth11 and WISH are essential for surface sensing, appressorium differentiation, and pathogenicity [16,17,18,19]. Genome-wide studies confirm that CFEM-GPCRs are expanded in phytopathogens and induced during infection [20]. In *Verticillium dahliae*, secreted CFEM effectors localize to the host plasma membrane and broadly suppress immunity; they can be classified into Asp- or Asn-types based on iron-binding residues, potentially separating iron acquisition from immune suppression roles [21]. Another *V. dahliae* CFEM effector, VdTRP, relies on membrane targeting for cytotoxicity, which can be neutralized by host apoplastic chitinase-like 1 [22]. Even in obligate biotrophic rust fungi, CFEM-containing candidates have been identified, such as in *Puccinia triticina* [23], and the stripe rust effector PstCFEM1 suppresses immunity and is required for full virulence [24]. Beyond these systems, CFEM proteins have been implicated in virulence and host immunity modulation across a broad range of other pathogens, including species of *Colletotrichum*, *Fusarium*, *Alternaria*, *Neofusicoccum*, *Marssonina*, and *Neostagonosporella*, highlighting their conserved yet adaptable role in fungal pathogenicity [25,26,27,28,29,30,31]. In particular, CFEM-containing effectors from various phytopathogens have been demonstrated to actively manipulate host programmed cell death (PCD), either eliciting or suppressing it, to facilitate infection [21,26,27].

The three-dimensional structure of CFEM proteins has been pivotal in understanding their functional mechanisms. The first solved CFEM domain, from *C. albicans* Csa2, adopts a unique helical-basket fold stabilized by four disulfide bonds from its eight characteristic cysteines. Heme is bound within this structure via a specific aspartate residue that coordinates ferric (Fe^3+^) iron, a feature likely critical for its acquisition role [32]. CFEM proteins often function as effectors, secreted to manipulate host immunity. They typically carry conserved motifs, such as CFEM, LysM, or RXLR, that facilitate their function or delivery [33]. These effectors can target key immune components: CfEC12 from *Colletotrichum fructicola* competitively binds apple MdNIMIN2 to disrupt salicylic acid signaling [34]; a group of *F. graminearum* CFEM proteins interacts with the maize receptor ZmWAK17 to suppress immune-triggered cell death [35]; and MaCFEM85 from the insect pathogen *Metarhizium anisopliae* activates jasmonic acid defenses by interacting with alfalfa MsWAK16 [36]. Evolutionarily, the CFEM domain has expanded in pathogenic fungi through gene duplication and is often under positive selection, reflecting adaptation to hosts [2,37]. In the *F. solani* species complex, CFEM genes are notably expanded and upregulated during infection, underscoring their role in cross-kingdom pathogenicity [38].

*S. sclerotiorum* is a devastating necrotrophic fungal pathogen with an exceptionally broad host range, causing stem rot or white mold on hundreds of dicotyledonous plants, resulting in significant economic losses worldwide. As a model necrotroph, it rapidly kills host tissue via a combination of lytic enzymes, oxalic acid, and effector proteins, facilitating nutrient acquisition from dead cells. Comparative secretome analyses between *S. sclerotiorum* and the related necrotroph *B. cinerea* have revealed important insights into their infection strategies. Notably, a key distinction was observed: while the predicted secretome of *B. cinerea* lacks proteins containing CFEM or LysM domains, that of *S. sclerotiorum* includes CFEM-containing proteins [15]. This finding suggests that CFEM proteins may represent a unique component of *S. sclerotiorum*’s virulence arsenal, potentially playing roles absent in *B. cinerea*. Despite the identification of CFEM candidates in its secretome and their implied importance, a comprehensive genome-wide identification and characterization of the CFEM protein family in *S. sclerotiorum* is entirely lacking.

To bridge this knowledge gap, this study aims to perform the first systematic and functionally oriented analysis of the CFEM protein family in *S. sclerotiorum*. We conducted a genome-wide identification of all *SsCFEM* genes, followed by a comprehensive in silico characterization of their sequence and structural features, phylogenetic relationships, and expression patterns during key life cycle stages. Importantly, to move beyond prediction and assign potential biological roles, we experimentally characterized the function of the candidate effectors using a heterologous plant expression system, specifically testing their ability to modulate host cell death. This integrated approach provides not only a foundational resource but also direct functional insights into the SsCFEM family, pinpointing key candidates involved in pathogenicity and host interaction for future mechanistic studies.

## 2. Results

### 2.1. Genome-Wide Identification and General Characteristics of the SsCFEM Protein Family

A comprehensive search of the *S. sclerotiorum* strain 1980 UF-70 genome, combining BLASTP+ 2.17.0 with the CFEM domain HMM profile and subsequent SMART validation, identified thirteen genes encoding proteins containing a bona fide CFEM domain. These were designated SsCFEM1 to SsCFEM13. Analysis of their primary sequences (Table S1) revealed substantial heterogeneity in key physicochemical properties (Table 1). The proteins varied considerably in size, with amino acid lengths ranging from 119 (SsCFEM10) to 995 (SsCFEM12), corresponding to predicted molecular weights between 12.25 kDa and 91.69 kDa. Their theoretical isoelectric points (pI) spanned a broad spectrum from acidic (pI 3.45 for SsCFEM2) to alkaline (pI 9.42 for SsCFEM12), suggesting diverse electrostatic properties that may influence subcellular localization and interaction partners. Stability predictions classified five proteins (SsCFEM4, SsCFEM5, SsCFEM6, SsCFEM9, and SsCFEM13) as stable (instability index below 40) and eight as unstable. Furthermore, the grand average of hydropathicity (GRAVY) calculations indicated that ten members are predominantly hydrophobic (GRAVY > 0), while SsCFEM2, SsCFEM11, and SsCFEM12 are hydrophilic (GRAVY < 0), hinting at potential differences in solubility and membrane association.

### 2.2. Analysis of Subcellular Localization and Effector Potential of SsCFEM Proteins

Bioinformatic tools were employed to predict the cellular fate and potential functional roles of the SsCFEM proteins (Table S1). Signal peptide prediction using SignalP-4.1 indicated that ten proteins possess a cleavable N-terminal signal peptide, targeting them to the secretory pathway (Table 2). Transmembrane helix analysis with TMHMM-2.0 revealed that eight of these secreted candidates lacked transmembrane domains and were thus classified as putative secreted proteins. The remaining five proteins were predicted to contain one or more transmembrane helices, suggesting they may be membrane-associated. Among the candidate secreted proteins, six were predicted to contain a glycosylphosphatidylinositol (GPI) anchor modification site via the big-PI Fungal Predictor, which could facilitate their attachment to the plasma membrane or cell wall. Notably, effector prediction using EffectorP 2.0 identified three of the secreted candidates—SsCFEM9, SsCFEM10, and SsCFEM13—as high-confidence fungal effectors, strongly implicating them in host–pathogen interactions.

**Figure 1:**
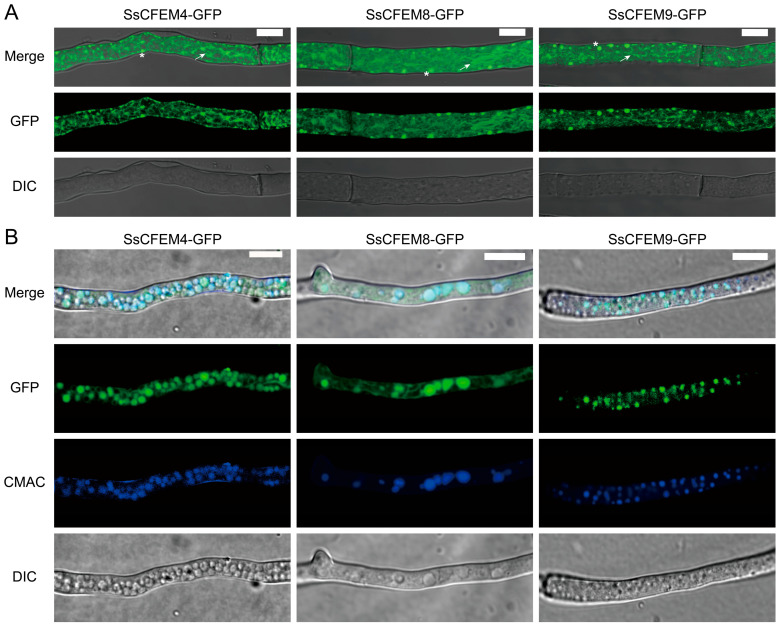
Subcellular localization of SsCFEM4, SsCFEM8, and SsCFEM9 in *S. sclerotiorum*. Fluorescence microscopy images show the localization of SsCFEM-GFP fusion proteins in hyphae of *S. sclerotiorum*. Stable transformants were generated by PEG-mediated protoplast transformation using linearized DNA fragments carrying the respective SsCFEM-GFP fusion construct and a G418 resistance marker, followed by selection on PDA medium containing 100 µg/mL G418. Mycelial plugs from genetically stable strains were cultured on PDA plates for 36 h and observed using laser scanning confocal microscopy (Nikon E100, Tokyo, Japan). (**A**) All three proteins display a cytoplasmic distribution characterized by a fine reticular network (indicated by arrows) and numerous punctate structures (marked by asterisks). The reticular pattern suggests association with the endoplasmic reticulum, whereas the puncta may correspond to vesicles, lysosomes, or vacuolar compartments. Scale bars = 10 µm. (**B**) The vacuoles were stained with 10 mM CellTracker™ Blue CMAC (7-amino-4-chloromethylcoumarin, Invitrogen) for 45 min at room temperature in the dark and visualized using confocal microscopy. Green fluorescence indicates the localization of CFEM protein, and blue fluorescence represents the vacuole marker. Scale bars = 10 µm.

To experimentally validate these predictions, we examined the subcellular localization of SsCFEM4, SsCFEM8, and SsCFEM9 via GFP fusion in *S. sclerotiorum*. Fluorescence microscopy revealed that all three proteins localized to the cytoplasm, exhibiting a composite pattern of a fine reticular network alongside numerous punctate structures (Figure 1A). Morphologically, this pattern is suggestive of an association with the endoplasmic reticulum (reticular network) and vesicles/lysosomes/vacuoles (puncta). To further confirm their subcellular localization, we stained the strain with the vacuole-specific dye CMAC and the mitochondrial marker CMXR. All three CFEM proteins were found to co-localize with CMAC, verifying their vacuolar localization (Figure 1B). In contrast, no co-localization was detected with the mitochondrial marker CMXR, suggesting that these proteins do not localize to mitochondria (Figure S1). Notably, the observed intracellular distribution for SsCFEM8 and SsCFEM9 contrasts with their primary bioinformatic prediction as extracellular proteins (Table 2), indicating potential retention within the secretory pathway or additional intracellular functions. For SsCFEM4, the observed pattern partially aligns with its predicted localization to the membrane and endomembrane compartments.

### 2.3. Conserved Motif Distribution and Cysteine Pattern Within the CFEM Domain

**Figure 2:**
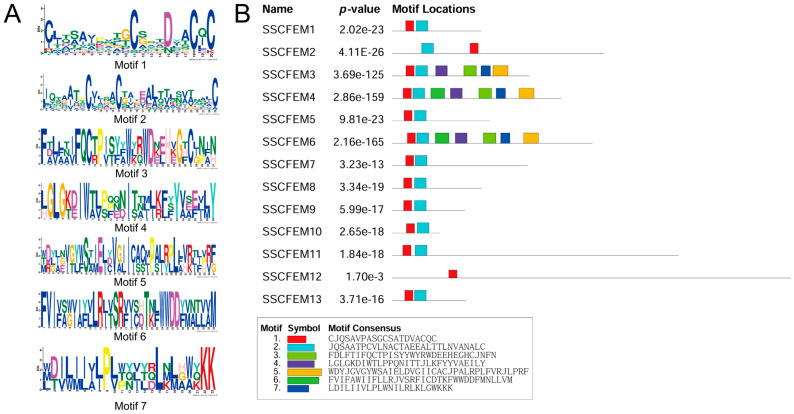
Conserved motif analysis of the SsCFEM proteins in *S. sclerotiorum*: (**A**) Sequence logos of the seven conserved motifs (Motifs 1–7) identified by MEME Suite analysis, depicting the amino acid conservation at each position. (**B**) Schematic representation of the motif distribution across the 13 SsCFEM proteins. Each motif is represented by a colored box as indicated. Motif 1 is present in all members and corresponds to the core region of the CFEM domain. Motif 2 is conserved in all members except SsCFEM12. Subgroups of proteins (SsCFEM3, SsCFEM4, and SsCFEM6) share specific combinations of additional motifs (Motifs 3–7), suggesting potential functional similarities or cooperation.

**Figure 3:**
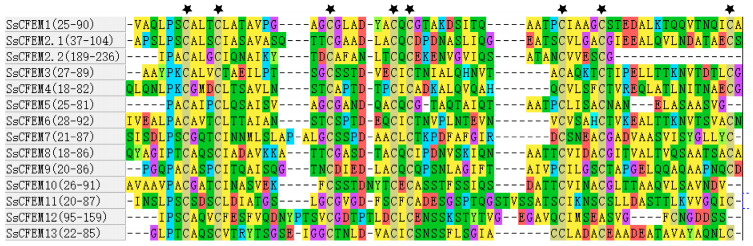
Multiple sequence alignment of the CFEM domains from *S. sclerotiorum*. Multiple sequence alignment was performed on the 14 CFEM domains identified from 13 SsCFEM proteins (Table S2) (with SsCFEM2 containing two domains, designated SsCFEM2.1 and SsCFEM2.2) using the ClustalW 2.0 algorithm in MEGA12. The alignment reveals a conserved pattern of cysteine residues at fixed positions. Eight cysteine residues (marked with asterisks) are strictly conserved in the majority of members. However, three domains—SsCFEM2.2 (the second domain of SsCFEM2), SsCFEM5, and SsCFEM10—lack one conserved cysteine in the C-terminal region of the domain. The SsCFEM12 domain lacks two of the typically conserved cysteines. The protein identifiers and the position of the aligned CFEM domain within the full-length sequence are indicated.

To uncover conserved sequence elements beyond the canonical domain, a motif analysis of the full-length SsCFEM proteins (Table S1) was conducted using the MEME Suite. This identified seven conserved motifs (Motifs 1–7) (Figure 2A). Their distribution across the family revealed distinct patterns (Figure 2B). Motif 1 was present in all members and corresponds to the core region of the CFEM domain. Motif 2 was conserved in all members except SsCFEM12, suggesting it may represent an auxiliary functional element critical for most family members. Specific subgroups of proteins, notably SsCFEM3, SsCFEM4, and SsCFEM6, shared a common set of additional motifs (Motifs 3–7), implying closer functional relatedness or cooperation. A detailed multiple sequence alignment of the isolated CFEM domains highlighted the hallmark cysteine pattern (Figure 3). Eight cysteine residues were strictly conserved at fixed positions in the majority of members, which are known to form disulfide bonds stabilizing the domain structure. However, deviations were observed: three domains—the second CFEM domain of SsCFEM2 (SsCFEM2.2), along with SsCFEM5 and SsCFEM10—lacked one conserved cysteine at the C-terminal region, while SsCFEM12 lacked two. These cysteine deficiencies suggest potential structural and functional diversification within the family.

### 2.4. Predicted Tertiary Structure and Phylogenetic Relationships

**Figure 4:**
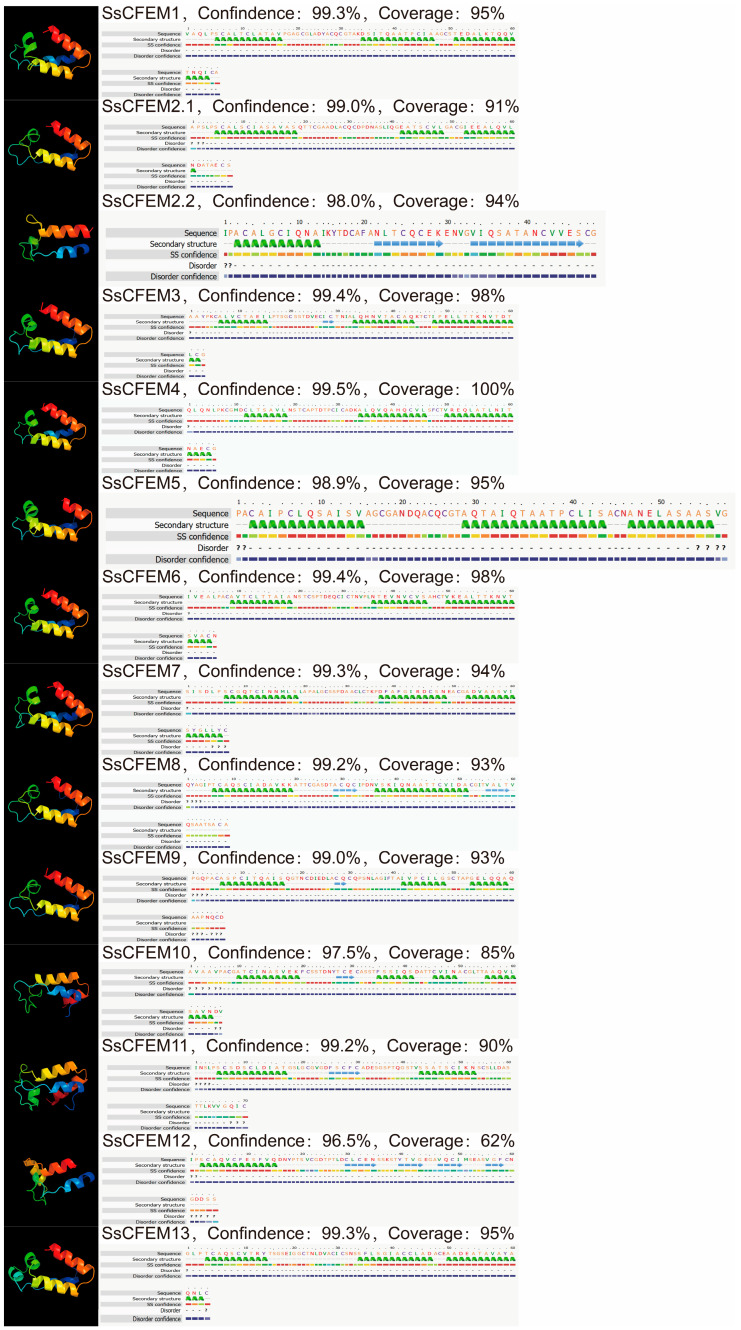
Predicted tertiary structural model of the CFEM domain in the SsCFEM protein. Structural models were generated for the 13 SsCFEM domains using Phyre2 with the crystal structure of the *C. albicans* Csa2 CFEM domain (PDB: 4Y7S) as a template. All models were predicted with >95% confidence and exhibit the characteristic “helix-basket” fold of the canonical CFEM domain. The core disulfide bond network, stabilized by four pairs of conserved cysteine residues as seen in Csa2, is largely preserved in the SsCFEM models. The model for SsCFEM12 shows notably lower coverage (62%) and a less ordered C-terminal region, consistent with its absence of two conserved cysteines identified in the sequence alignment. All amino acid sequences of *S. sclerotiorum* CFEM-containing proteins are from Supplementary Table S2. In the sequence alignment, colors represent amino acid properties: yellow indicates small/polar residues (A, S, T, G, P); green indicates hydrophobic residues (M, I, L, V); red indicates charged residues (K, R, E, N, D, H, Q); and purple indicates aromatic residues and cysteine (W, Y, F, C). Dashes (‘-’) denote gaps introduced to optimize the alignment. Question marks (‘?’) indicate positions with uncertain alignment or residues that could not be confidently assigned. In the structural model, green helices represent α-helices, and blue arrows represent β-strands.

**Figure 5:**
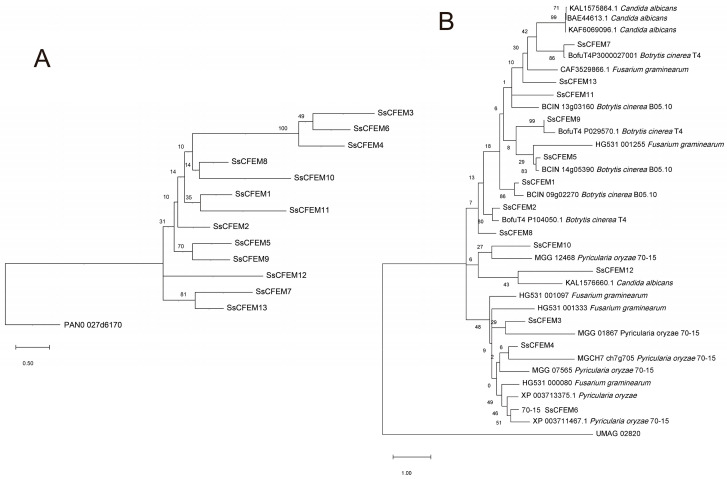
Phylogenetic analysis of the SsCFEM proteins: (**A**) Intraspecific phylogenetic tree of the 13 SsCFEM proteins from *S. sclerotiorum*. The tree was constructed using the maximum likelihood method in MEGA12 with the JTT+G model. Numbers at nodes represent bootstrap support values from 1000 replicates. An evolutionary tree was built with the CFEM protein PAND_027d6170 of *U. maydis* serving as the outgroup, together with all CFEM proteins from *S. sclerotiorum*. Within the ingroup, CFEM proteins did not cluster into a single, highly supported clade but were distributed across multiple fine-scale branches. Notably, conserved motif analysis revealed that members harboring multiple motifs (SsCFEM3, SsCFEM4, and SsCFEM6) clustered together into a compact, highly supported subclade, implying potential functional similarity among these proteins. The scale bar below the branch lengths represents the evolutionary distance estimated based on the model used, corresponding to the average number of substitutions per site. (**B**) The phylogenetic tree was constructed using the maximum likelihood method with the WAG+G model in MEGA 12, with the UMAG_02820 protein from *U. maydis* selected as the outgroup. Interspecific phylogenetic tree including CFEM proteins from *S. sclerotiorum* and selected fungal species (*M. oryzae*, *B. cinerea*, *F. graminearum*, and *C. albicans*). The SsCFEM proteins do not form a monophyletic group but are distributed across different evolutionary clusters. A subset of SsCFEMs clusters with *B. cinerea* CFEMs with strong support (>80% bootstrap), suggesting a common ancestral origin potentially related to pathogenicity. The remaining SsCFEMs are dispersed in other clades, indicating that the family has undergone multiple independent gene duplications and lineage-specific expansions, possibly acquiring specialized functions adapted to the necrotrophic lifestyle of *S. sclerotiorum*. The scale bar below the branch lengths represents the evolutionary distance estimated based on the model used, corresponding to the average number of substitutions per site. All amino acid sequence data for the proteins used are from Supplementary Tables S1 and S3.

Homology modeling using Phyre2, with the *C. albicans* Csa2 (PDB: 4Y7S) structure as a template, generated high-confidence (>95%) three-dimensional models for all SsCFEM proteins (Figure 4). All models adopted the characteristic “helix-basket” fold definitive of the CFEM domain. The core structural network, stabilized by four pairs of conserved cysteine-derived disulfide bonds as seen in Csa2, was largely recapitulated in the SsCFEM models. The model for SsCFEM12 showed notably lower sequence coverage (62%) and a less ordered C-terminal region, consistent with its absence of two key cysteines identified in the alignment. The topology of the rooted tree indicates that the CFEM protein family of *S. sclerotiorum* forms a distinct monophyletic group, which exhibits a sister-group relationship with the CFEM protein PAND_027d6170 from *Ustilago maydis* (Figure 5A). This suggests an early evolutionary divergence following their speciation from a common ancestor. Within the ingroup, CFEM proteins did not cluster into a single, highly supported clade but were distributed across multiple fine-scale branches. Notably, conserved motif analysis revealed that members harboring multiple motifs (SsCFEM3, SsCFEM4, and SsCFEM6) clustered together into a compact, highly supported subclade (Figure 2B), implying potential functional similarity among these proteins. A broader interspecific phylogeny including CFEMs from other fungi revealed that SsCFEM proteins are polyphyletic, dispersing across different evolutionary clusters rather than forming a single monophyletic group (Figure 5B). A subset of SsCFEMs clustered with strong support (>80% bootstrap) alongside CFEMs from the related necrotroph *B. cinerea*, indicating a shared ancestral origin potentially linked to pathogenicity. The dispersal of other SsCFEM members suggests that the family has undergone multiple independent duplications and lineage-specific expansion events in *S. sclerotiorum*.

### 2.5. Genomic Architecture

**Figure 6:**
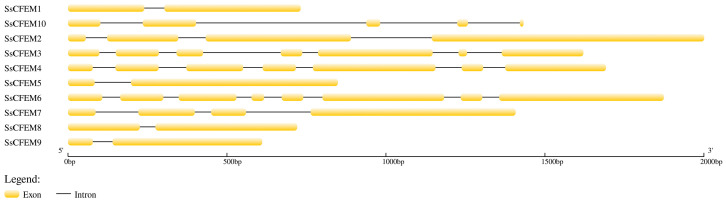
Gene structure of the SsCFEM family members. The schematic diagram of gene structure was generated using the Gene Structure Display Server 2.0 online tool. Exons are represented by yellow rectangles, introns by black lines. The number of exons varies from 2 to 8 among the members. This structural diversity correlates clearly with the phylogenetic clades defined in Figure 5A: Several intron-containing members (SsCFEM3, SsCFEM4, and SsCFEM6) formed a cluster in the phylogenetic tree (Figure 5A), constituting a well-supported monophyletic clade. This distinct divergence in gene organization provides genomic evidence supporting the functional differentiation and independent evolutionary history of the two subfamilies. All genomic and coding sequence (CDS) data for *S. sclerotiorum* are from Supplementary Tables S4 and S5.

Analysis of gene structure using GSDS 2.0 revealed a significant correlation between exon-intron organization and phylogenetic clustering as well as conserved motifs (Figure 6). Notably, several intron-containing members (SsCFEM3, SsCFEM4, and SsCFEM6) formed a cluster in the phylogenetic tree (Figure 5A), constituting a well-supported monophyletic clade. This distinct genomic structural feature provides further support for the evolutionary relationships inferred from the phylogeny.

### 2.6. Expression Dynamics During In Vitro Growth and Early Host Colonization

**Figure 7:**
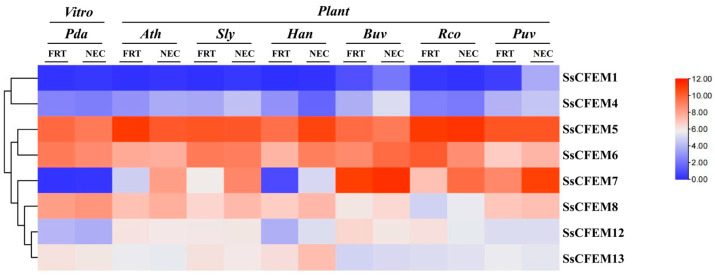
Expression patterns of *SsCFEM* genes during in vitro growth and early colonization of different host plants. Gene expression profiles were derived from a published transcriptomic dataset (NCBI SRA: GSE159792), which includes *S. sclerotiorum* samples from in vitro mycelial zones (center and apex) and from early infection stages on six host plants. Of the 13 family members, expression data were available for eight *SsCFEM* genes in this dataset. Their FPKM values were extracted and visualized as a hierarchical clustered heatmap using TBtools-II. Each row represents an *SsCFEM* gene, and each column represents a biological condition. Expression levels are normalized and color-coded (see scale), with red indicating higher expression and blue indicating lower expression. The analysis reveals distinct expression dynamics: most genes (5 out of 8, including *SsCFEM1* and *SsCFEM8*) are upregulated in one or more host plants compared to in vitro conditions. *SsCFEM1* shows constitutively high expression in most samples. *SsCFEM8* exhibits a marked induction in all infected hosts, with peak expression in *Beta vulgaris* and consistently higher expression in the mycelial apex than in the center across all sample types. In contrast, *SsCFEM13* is generally downregulated in planta. The differential expression patterns among hosts and between mycelial zones suggest host-specific and spatially regulated roles for these *SsCFEM* genes during infection.

Transcriptomic analysis of publicly available RNA-Seq data (GSE159792) (https://www.ncbi.nlm.nih.gov/geo/query/acc.cgi?acc=GSE159792 (accessed on 9 December 2025)) elucidated the expression patterns of eight detectable *SsCFEM* genes across different growth and infection contexts (Figure 7). As the gene annotation for the GSE159792 dataset contains only eight of the thirteen *SsCFEM* genes—excluding *SsCFEM2*, *3*, *9*, *10*, and *11*—our expression analysis was restricted to those eight detectable members. During in vitro mycelial growth, several genes displayed spatial regulation, with significantly higher expression in the actively growing apex compared to the colony center; this pattern was most pronounced for *SsCFEM8*. During the early colonization stage across six different host plants, the majority of detected genes (5 out of 8) were upregulated in planta compared to in vitro conditions, consistent with a role during infection. *SsCFEM8* exhibited the most robust and consistent induction, being upregulated in all tested hosts, with peak expression observed during infection of sugar beet (*Beta vulgaris*). Conversely, *SsCFEM13* was generally downregulated during plant infection. The expression profiles of *SsCFEM* genes also showed variation depending on the host plant species, indicating that *S. sclerotiorum* modulates its CFEM effector repertoire in a host-specific manner. The combined expression data highlight *SsCFEM8* as a prime candidate for a key virulence factor involved in host penetration and/or early colonization.

### 2.7. Functional Validation of SsCFEM Proteins as Cell Death Suppressors

**Figure 8:**
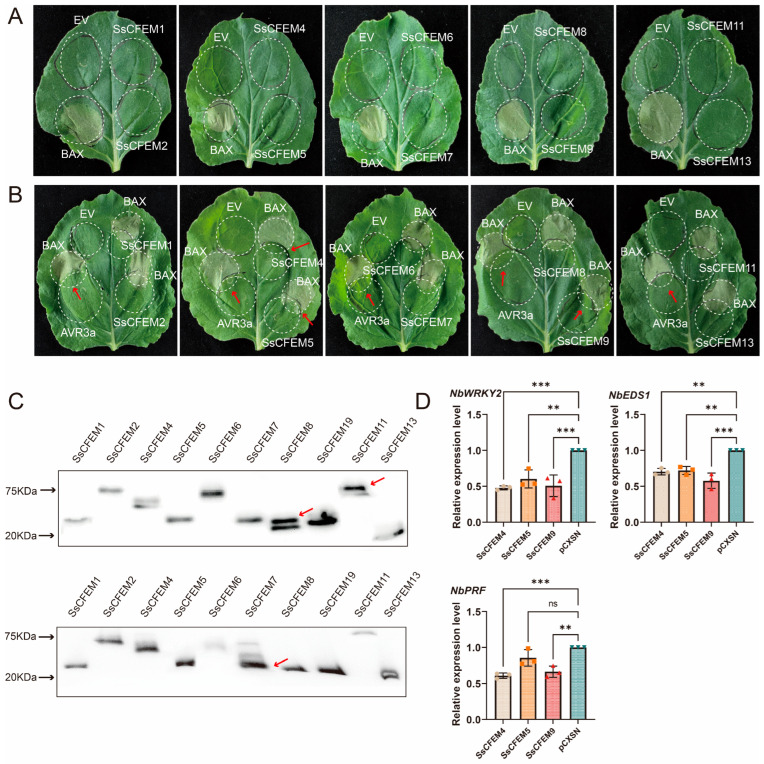
Functional validation of SsCFEM proteins as modulators of programmed cell death in *N. benthamiana* via transient expression: (**A**) Cell death induction assay. The left half of each leaf served as an internal control: infiltration with the empty vector pCXSN (EV, negative control) in the upper left and pro-apoptotic BAX [39] (positive control) in the lower left. The white dotted circles in the figure delineate the range of *A. tumefaciens* infiltration. Symptoms were monitored for 5 days. None of the ten tested SsCFEM proteins induced cell death when expressed alone. (**B**) Cell death suppression assay. Leaves were first infiltrated with *A. tumefaciens* carrying EV (upper left, negative control), the known suppressor AVR3a [40] (lower left, positive control), or SsCFEM constructs (right half). After 24 h, the same areas were reinfiltrated with BAX to trigger cell death. The white dotted circles indicate the areas infiltrated with *A. tumefaciens* suspensions. The red arrow marks the junction between two infiltration zones, where the absence of cell death indicates suppression of BAX-induced cell death. SsCFEM5 showed weak suppression, whereas SsCFEM4 and SsCFEM9 potently suppressed BAX-induced cell death. (**C**) Western blot analysis to determine whether SsCFEM is successfully expressed during the induction of cell necrosis and suppression of cell necrosis. The upper panel shows the expression of SsCFEM proteins during cell death induction, where all 10 SsCFEM proteins were successfully detected using an anti-HA antibody. The red arrow highlights the specific SsCFEM protein band amidst background noise from non-specific bands.The lower panel shows the expression of SsCFEM proteins during cell death suppression, where the same 10 effector proteins were successfully detected using an anti-HA antibody. The molecular weights of the SsCFEM proteins fused with the HA tag range from approximately 21 kDa to 80 kDa. The estimated sizes are as follows: SsCFEM1-HA (~26 kDa), SsCFEM2-HA (~59 kDa), SsCFEM4-HA (~48 kDa), SsCFEM5-HA (~25 kDa), SsCFEM6-HA (~56 kDa), SsCFEM7-HA (~38 kDa), SsCFEM8-HA (~26 kDa), SsCFEM9-HA (~21 kDa), SsCFEM11-HA (~80 kDa), and SsCFEM13-HA (~21 kDa). Multiple bands were observed, and the red arrows indicate the specific target bands. (**D**) qRT-PCR analysis of PTI marker gene expression in *N. benthamiana*. Relative transcript levels of *NbWRKY2*, *NbEDS1*, and *NbPRF* were measured in leaves expressing SsCFEM4, SsCFEM5, SsCFEM9, or the empty vector (pCXSN) at 24 h post-infiltration. Expression was normalized to a tobacco housekeeping gene using the 2^−ΔΔCt^ method. All three marker genes were significantly downregulated by SsCFEM4, SsCFEM5, and SsCFEM9 compared to the empty vector control, with SsCFEM4 and SsCFEM9 showing stronger repression, consistent with the cell death suppression assay. Data represent means ± SD from three biological replicates. Asterisks indicate significant differences compared to the control (** *p* < 0.01, *** *p* < 0.001; ns, not significant; Student’s *t*-test).

To experimentally assess their functional role, we performed transient expression assays in *Nicotiana benthamiana*. Ten of the thirteen *SsCFEM* genes were successfully cloned and expressed. Initial screens showed that none induced cell death when expressed alone (Figure 8A). However, in a suppression assay co-expressing each with the pro-apoptotic protein BAX, SsCFEM4 and SsCFEM9 potently inhibited BAX-induced cell death, while SsCFEM5 showed weaker activity (Figure 8B). The remaining seven members had no significant effect. Concurrently, Western blot analysis using an anti-HA antibody was performed to detect the expression of SsCFEM proteins in all experimental groups, thereby further validating the reliability of the cell death induction and suppression assays mediated by SsCFEM from *S. sclerotiorum*. The results demonstrated that SsCFEM proteins were successfully detected in all treatment groups (Figure 8C), confirming the credibility of the cell death induction and suppression experiments.

To verify the accuracy of the cell death suppression assay, RNA was extracted from *N. benthamiana* leaves infiltrated with *Agrobacterium tumefaciens* cultures harboring SsCFEM4, SsCFEM5, SsCFEM9, or the empty vector pCXSN. Following reverse transcription, quantitative real-time PCR (qRT-PCR) was performed to assess the expression of PTI pathway marker genes. The results showed that the transcript levels of *NbWRKY2* (GeneID: 107780258), *NbEDS1* (GeneID: 107782626), and *NbPRF* (GeneID: 107782528) were significantly downregulated in leaves expressing SsCFEM4, SsCFEM5, or SsCFEM9 compared to the pCXSN control. Notably, SsCFEM4 and SsCFEM9 induced a more pronounced downregulation of these PTI marker genes than SsCFEM5, a trend consistent with the observed suppression of cell death.

This functional validation directly supports and extends our bioinformatic predictions: SsCFEM9, a predicted effector (Table 2), is confirmed as a cell death suppressor. Interestingly, SsCFEM4, not predicted as an effector, also exhibits this activity.

## 3. Discussion

This study provides the first genome-wide analysis of the CFEM protein family in the broad-host-range necrotroph *S. sclerotiorum*. We identified 13 SsCFEM proteins exhibiting substantial diversity in sequence, structure, and predicted subcellular localization. Their complex phylogenetic and expression patterns suggest nuanced regulatory mechanisms and potential adaptation to host infection. Notably, functional screening revealed that specific members, such as SsCFEM4 and SsCFEM9, act as suppressors of plant cell death, highlighting their likely role in modulating host immunity during pathogenesis.

### 3.1. Diversity and Predicted Functional Roles of SsCFEM Proteins

The identification of 13 CFEM proteins in *S. sclerotiorum* places it among fungi with a moderate number of such domains, consistent with patterns observed in other phytopathogens where CFEM copy number can correlate with pathogenic lifestyle [2]. The high sequence and physicochemical heterogeneity within the family suggest functional diversification (Table 1). Our prediction that a subset of SsCFEMs are canonical secreted proteins lacking transmembrane domains aligns with the well-established role of CFEM proteins as extracellular effectors in other fungi. For instance, proteins such as Rbt5 and Pga7 in *C. albicans* function as hemophores involved in nutrient acquisition [4], whereas other CFEM family members, including certain proteins in *V. dahliae*, have been characterized as effectors that suppress host immunity [21]. The prediction of GPI-anchor modification sites in several secreted SsCFEMs is particularly noteworthy (Table 2). In *C. albicans*, GPI-anchored CFEM proteins like Rbt5 are integral to cell wall architecture and heme-iron acquisition [6]. Similarly, in *B. cinerea*, the GPI-anchored CFEM protein BcCFEM1 contributes to virulence [13]. This suggests that GPI-anchoring may be a conserved mechanism for localizing CFEM domains to the fungal cell surface to mediate interactions with the host environment, potentially including nutrient scavenging or adhesion.

The identification of three SsCFEMs (SsCFEM9, 10, and 13) as high-confidence effectors by EffectorP further supports their potential involvement in manipulating host physiology (Table 2). This is reminiscent of CFEM effectors in other plant pathogens, such as PstCFEM1 from wheat stripe rust, which suppresses host immunity [24], and a suite of CFEM effectors in *Fusarium* species that inhibit plant cell death [26,27]. The presence of proteins with multiple transmembrane helices (e.g., SsCFEM11, 12) defines another subfamily, possibly functioning as membrane-bound receptors or transporters (Table 2). The CFEM-containing GPCR Pth11 in *M. oryzae* is a key precedent, essential for surface sensing and pathogenicity [16,17]. Although our predictions did not strongly indicate GPCR domains for these transmembrane SsCFEMs, they may represent a divergent class of membrane-integral CFEM proteins with undiscovered signaling or transport functions.

### 3.2. Structural Insights and Evolutionary Trajectory

The conservation of the core “helical-basket” fold, as predicted by AlphaFold2 and confirmed by high model confidence (Figure 4), underscores the structural unity of the CFEM domain across kingdoms, from human pathogens like *C*. *albicans* [32] to plant pathogens like *S. sclerotiorum*. However, variations in the canonical eight-cysteine pattern, observed in several SsCFEMs (Figure 3), indicate potential structural plasticity. Such variations have been noted in CFEM families of other fungi, like *F. graminearum* [41], and may lead to altered disulfide bonding, affecting ligand binding specificity or protein stability. The markedly lower structural coverage for SsCFEM12, which lacks two cysteines, strongly suggests a distinct structural conformation, possibly reflecting a specialized function (Figure 4). Phylogenetic analysis provides crucial insights into the evolutionary history of this family. The topology of the rooted tree reveals that the CFEM protein family in *S*. *sclerotiorum* does not exhibit deep phylogenetic divergence, but instead forms multiple shallow, poorly supported branches radiating from a common ancestral node (Figure 5A). This pattern of limited diversification suggests that the family may have undergone strong functional constraints during subsequent evolution, leading to conserved sequence and functional characteristics. The non-monophyletic distribution of SsCFEMs in the interspecific tree suggests a complex evolutionary history (Figure 5B). The clustering of some SsCFEMs with CFEMs from the necrotroph *B*. *cinerea* with high support implies shared ancestry and possibly conserved functions related to necrotrophy, such as host cell death manipulation or detoxification of plant defenses. The scattering of other SsCFEMs across the tree likely results from lineage-specific gene duplication and expansion, a driving force in the evolution of pathogen effector repertoires [37]. This pattern of both conservation and lineage-specific innovation is common in fungal pathogenicity factor families and allows for adaptation to specific hosts or niches.

### 3.3. Expression Dynamics Suggest Context-Specific Functions

The expression profiling during plant infection offers the most direct functional clues (Figure 7). The widespread upregulation of *SsCFEM* genes in planta, especially the strong and consistent induction of *SsCFEM8* across all hosts, strongly implicates these genes in virulence. This pattern mirrors the induction of CFEM effector genes during infection in other pathosystems, such as *FsCFEMs* in *F. sacchari* [26] and *PstCFEM1* in wheat stripe rust [24]. The specific high expression of *SsCFEM8* in the mycelial apex, both in vitro and in planta, points to a role in hyphal tip growth, host surface sensing, or initial penetration—functions analogous to the CFEM-GPCR WISH in *M. oryzae* [17]. Conversely, the downregulation of *SsCFEM13* during infection suggests its primary role may be in saprophytic growth or development, highlighting functional specialization within the family. The host-specific variation in expression profiles further suggests that *S. sclerotiorum* employs a flexible strategy, modulating its CFEM arsenal to optimize interaction with different plant species.

### 3.4. Experimental Validation of Cell Death Suppression Activity

Our transient expression assays provide the first experimental evidence for the function of SsCFEM proteins as modulators of plant cell death, a cornerstone of plant defense and necrotrophic pathogenesis (Figure 8). The potent suppression of BAX-induced PCD by SsCFEM4 and SsCFEM9 aligns with the emerging paradigm of necrotrophic effectors that actively inhibit host immunity to facilitate colonization [39]. This finding is particularly significant for SsCFEM9, as it confirms its bioinformatic classification as a high-confidence effector (Table 2). The activity of SsCFEM4, which was not predicted as an effector by computational tools, underscores the complementary value of experimental screening in identifying functional candidates that may be missed by current algorithms.

The functional divergence observed—where only a subset of tested SsCFEMs showed suppressive activity—parallels their sequence, structural, and phylogenetic diversity (Figure 2, Figure 3 and Figure 5). It is plausible that cell death suppression is a specialized function acquired by specific members within Clades I and II, possibly through distinct molecular mechanisms. The failure to clone SsCFEM3, 10, and 12 under standard laboratory growth conditions is intriguing. Their functional characterization remains an important goal for future work.

The lack of cell death-inducing activity for any SsCFEM is noteworthy (Figure 8A). Unlike some necrotrophic effectors that directly elicit necrosis, these SsCFEM suppressors may function early during infection to dampen pattern-triggered immunity (PTI), creating a permissive environment for the fungus before the onset of widespread tissue necrosis driven by lytic enzymes and oxalic acid. This “stealth” strategy would be consistent with the early upregulation of several *SsCFEM* genes, including *SsCFEM8*, during host colonization (Figure 7).

### 3.5. Limitations and Future Perspectives

This study provides a robust predictive framework and initial functional validation for the SsCFEM family. Key limitations include reliance on a single reference strain, which may not capture the full allelic diversity of SsCFEMs across populations, and the use of transcriptomic data from early infection stages, potentially missing roles during later necrotrophic growth or sclerotial development. Furthermore, our functional assays are based on heterologous expression in a model plant. While informative, confirming these activities during actual infection by *S. sclerotiorum* through gene knockout/complementation is essential.

Future research should prioritize elucidating the molecular mechanisms by which SsCFEM4 and SsCFEM9 suppress cell death (Figure 8B) and determining the in planta role of the high-expression candidate SsCFEM8 (Figure 7). Gene knockout and complementation studies in *S. sclerotiorum* are essential to confirm their roles in virulence, host range, and specific pathogenic processes like oxalate production or tissue maceration. For transmembrane members like SsCFEM11/12, investigating their role in sensing or transport will be crucial (Table 2). An interesting direction for future research is to explore whether SsCFEM proteins, beyond their immune-modulating functions, may also participate in iron acquisition. In *C. albicans*, the CFEM proteins Rbt5 and Pga7 bind heme via a conserved aspartic acid (Asp) residue essential for iron scavenging [4]. Recent work in *V. dahliae* has shown that mutation of this residue can drive functional divergence from iron acquisition toward immune suppression [21]. Whether SsCFEM proteins possess this conserved residue and retain heme-binding capability remains to be determined. Biochemical studies are needed to determine if SsCFEMs bind heme or other ligands, as seen in *Candida* [3,6], which would link them to iron acquisition—a critical virulence factor even for necrotrophs. Furthermore, identifying host targets of the predicted effector SsCFEMs, following examples like the interaction between *F. graminearum* CFEMs and maize ZmWAK17 [35] or *C. fructicola* CfEC12 and apple MdNIMIN2 [34], will unravel the molecular mechanisms of immune suppression. For the uncloned members (SsCFEM3, 10, and 12), alternative strategies such as using induced RNA-seq guided cDNA synthesis from infected tissue should be employed to capture their transcripts and elucidate their functions.

## 4. Materials and Methods

### 4.1. Bacterial Strains, Plant Materials, Vectors, and Primers

The fungal strain *S. sclerotiorum* 1980 was used in this study. *N. benthamiana* plants were employed as the plant material. The vectors used included the plant expression vector pCXSN and the pNAH-ONG vector, which carries the *Olic* promoter and a green fluorescent protein (GFP) tag. All primers used are listed in Supplementary Table S6.

### 4.2. Genome-Wide Identification and Physicochemical Characterization

The genome sequence and annotation of *S. sclerotiorum* strain 1980 UF-70 were used as the basis for this study. CFEM domain-containing proteins were initially identified by performing a local BLASTP search against the *S. sclerotiorum* proteome using the hidden Markov model (HMM) profile of the CFEM domain (Pfam: PF05730) as the query, with an E-value cutoff of 1 × 10^−5^. All candidate protein sequences were subsequently validated for domain integrity using the SMART online database (https://smart.embl.de/smart/change_mode.cgi) (accessed on 15 November 2025). Proteins confirmed to contain a bona fide CFEM domain were designated SsCFEM1 to SsCFEM13. Their basic physicochemical properties, including amino acid number, molecular weight, theoretical isoelectric point (pI), instability index, and grand average of hydropathicity (GRAVY), were computed using the ExPASy ProtParam tool (https://web.expasy.org/protparam/) (accessed on 15 November 2025).

### 4.3. Sequence Alignment, Conserved Motif, and Gene Structure Analysis

Multiple sequence alignment of the CFEM domains extracted from the 13 SsCFEM proteins was performed using the ClustalW algorithm implemented in MEGA12 software (Table S2). For the discovery of conserved motifs across the full-length protein sequences, the MEME Suite (https://meme-suite.org/meme/doc/download.html) (accessed on 15 November 2025) was employed with the following parameters: maximum number of motifs to find = 10, minimum motif width = 6, and maximum motif width = 50 amino acids.

The exon–intron structures of the *SsCFEM* genes were illustrated using the Gene Structure Display Server 2.0 (https://gsds.gao-lab.org/Gsds_help.php) (accessed on 15 November 2025). Genomic DNA sequences and their corresponding coding sequences (CDSs) were obtained from the genome annotation file to generate schematic diagrams depicting the number, length, and arrangement of exons (represented as yellow rectangles) and introns (represented as black lines) (Tables S4 and S5). Since the coding sequences (CDSs) of *SsCFEM11 to SsCFEM13* were only available in the EMBL (https://www.ebi.ac.uk/) (accessed on 15 November 2025) database, and their non-coding regions could not be retrieved, the gene structure analysis in this study was limited to SsCFEM1 to SsCFEM10.

### 4.4. Prediction of Subcellular Localization and Effector Potential

The presence and cleavage site of N-terminal signal peptides were predicted using SignalP-4.1 (https://services.healthtech.dtu.dk/services/SignalP-4.1/) (accessed on 15 November 2025) with default settings for eukaryotes. Transmembrane helices were predicted using TMHMM-2.0 (https://services.healthtech.dtu.dk/services/TMHMM-2.0/) (accessed on 15 November 2025). Mitochondrial targeting peptides were predicted using TargetP 2.0 (https://services.healthtech.dtu.dk/services/TargetP-2.0/) (accessed on 15 November 2025). Potential glycosylphosphatidylinositol (GPI) anchor modification sites were predicted using the big-PI Fungal Predictor (https://mendel.imp.ac.at/gpi/fungi_server.html) (accessed on 15 November 2025). The potential of SsCFEM proteins to function as fungal effectors was assessed using EffectorP 3.0 (https://effectorp.csiro.au/) (accessed on 15 November 2025).

### 4.5. Phylogenetic Analysis

Two phylogenetic trees were constructed using the maximum likelihood (ML) method in MEGA12 software. First, a phylogenetic tree was constructed using the full-length sequences of the 13 SsCFEM proteins (Table S1), with the CFEM protein PAND_027d6170 from *U. maydis* as the outgroup (Table S3). Second, an interspecific tree was constructed by aligning the SsCFEM sequences with previously characterized CFEM protein sequences from representative fungi (*M. oryzae*, *B. cinerea*, *F. graminearum*, and *C. albicans*), which were retrieved from the NCBI database. For the phylogenetic tree of multiple species, we selected the UMAG_02820 protein from *U. maydis* as the outgroup. For both analyses, the best-fit amino acid substitution model (JTT+G for the intraspecific tree; WAG+G for the interspecific tree) was selected based on the lowest Bayesian Information Criterion (BIC) score. The robustness of tree topology was assessed by bootstrap analysis with 1000 replicates.

### 4.6. Three-Dimensional Structure Modeling

The three-dimensional (3D) structures of the SsCFEM domains were predicted using the protein homology modeling server Phyre2 (https://www.sbg.bio.ic.ac.uk/~phyre2/html/page.cgi?id=index) (accessed on 15 November 2025) in the intensive modeling mode. The crystal structure of the *C. albicans* Csa2 CFEM domain (PDB: 4Y7S) was used as the primary template. Models with confidence scores > 95% were selected for visualization. The final models were rendered to highlight secondary structure elements (α-helices as green cylinders, β-strands as blue arrows) and amino acid properties colored by type (small/polar: yellow; hydrophobic: green; charged: red; aromatic and cysteine: purple).

### 4.7. In Silico Expression Profiling

Publicly available RNA-Seq data under NCBI accession GSE159792 [42] were utilized to analyze *SsCFEM* gene expression. This dataset includes transcriptomes from *S. sclerotiorum* during in vitro mycelial growth (colony center and apex) and during early infection stages on six different host plants (*Arabidopsis thaliana*, *Beta vulgaris*, *Ricinus communis*, *Phaseolus vulgaris*, *Helianthus annuus*, and *Solanum lycopersicum*). Fragments Per Kilobase of transcript per Million mapped reads (FPKM) values for the *SsCFEM* genes present in the dataset were extracted. A hierarchical clustered heatmap was generated using TBtools-II to visualize expression dynamics across the different biological conditions.

### 4.8. Transient Expression Assays in N. benthamiana

The coding sequences of *SsCFEM* genes were cloned into the plant expression vector pCXSN. which provides an N-terminal HA tag under the control of the CaMV 35S promoter. The constructs were transformed into *A. tumefaciens* strain GV3101. Bacterial suspensions were prepared in infiltration buffer (10 mM MES, 20 g/L sucrose, 200 μM acetosyringone) and adjusted to OD600 = 1.0 for leaf infiltration in 4–5-week-old *N. benthamiana* plants.

To screen for cell death-inducing activity, leaves were infiltrated with *A. tumefaciens* suspensions carrying individual SsCFEM constructs. The empty vector served as a negative control, and BAX alone served as the positive control for cell death induction. For cell death suppression assays, leaves were first infiltrated with *A. tumefaciens* carrying an SsCFEM construct. After 24 h, an adjacent site was infiltrated with *A. tumefaciens* carrying BAX to create an overlapping zone, which was then assessed for suppression of BAX-induced cell death. Controls for this assay included the empty vector (negative control) and the co-infiltration of BAX with the known suppressor AVR3a (positive control for suppression). All infiltrated plants were kept under low-light conditions for 24 h and then transferred to normal light. Cell death symptoms were visually monitored daily for up to 5 days post-infiltration. All experiments included at least three biological replicates and were repeated twice independently. Total proteins were extracted from the infiltrated zones of tobacco leaves and analyzed by Western blot with an anti-HA antibody to determine the expression of HA-tagged SsCFEM proteins.

### 4.9. Analysis of PTI Marker Gene Expression

*A. tumefaciens* strains harboring the SsCFEM-pCXSN recombinant plasmid or the pCXSN empty vector were resuspended in infiltration buffer to an optical density and adjusted to OD600 = 1.0. The bacterial suspensions were then infiltrated into the abaxial side of the leaves of *N. benthamiana*. The empty vector strain served as a negative control. At least three biological replicates were performed for each treatment.

Leaf tissue samples from the infiltrated zones were collected at 24 h post-infiltration. Total RNA was extracted using a plant RNA extraction kit, and its concentration was determined. After DNase I digestion to remove genomic DNA contamination, 1 μg of total RNA was reverse-transcribed into first-strand cDNA using a reverse transcription kit.

Quantitative real-time PCR (qRT-PCR) was performed using SYBR Green Premix on a real-time PCR detection system, with the cDNA as template. The expression levels of the target PTI marker genes (*PRF*, *EDS1*, and *WRKY2*) were examined, and a tobacco housekeeping gene was used as an internal control. Three technical replicates were analyzed for each sample. Relative gene expression levels were calculated using the 2^−ΔΔCt^ method to compare expression differences between the treatment and control groups. All the primers used in this study are listed in Table S6. The reverse transcription kit (Cat. No. R333-C1, Version 24.1) and SYBR Green qPCR mix (Cat. No. Q312, Version 23.1) were purchased from Nanjing Vazymc, Nanjing, China

### 4.10. Subcellular Localization Analysis

To determine the subcellular localization of SsCFEM proteins, GFP-fusion constructs were expressed in *S. sclerotiorum* while retaining their native signal peptides. The coding sequence (CDS) of each *SsCFEM* gene was amplified from wild-type strain 1980 cDNA and cloned into the SpeI-digested pNAH-ONG vector using a seamless cloning kit, generating a C-terminal GFP fusion plasmid with a G418 resistance marker. After sequence verification, the recombinant plasmid was used as a template to amplify a linearized fragment with primers pmtF/pmtR. This linear DNA was then introduced into *S. sclerotiorum* protoplasts via PEG-mediated transformation. Following transformation, the mixture was incubated in antibiotic-free RM medium for 12–16 h at 22 °C, after which transformants were selected by overlaying with SRM medium containing 100 µg/mL G418. Positive transformants were subcultured 3–4 times on G418-containing PDA to ensure genetic stability.

For microscopy, mycelial plugs from stable strains were examined using a laser scanning confocal microscope. Fluorescence distribution was recorded from at least three independent fields per strain. The GFP signals were observed using 488 nm light. The vacuoles were stained with 10 mM CellTracker™ Blue CMAC (7-amino-4-chloromethylcoumarin, Invitrogen) for 45 min at room temperature in the dark and observed with the DAPI channel using 405 nm light. The mitochondria were stained with 1 mM MitoTracker™ Red CMXR (Chloromethyl-X-rosamine, Invitrogen, Source:Thermo Fisher Scientific Eugene USA) for 45 min at room temperature in the dark and observed with the RFP channel using 561 nm light.

## 5. Conclusions

In summary, our integrated study deciphers the CFEM protein repertoire of *S. sclerotiorum* and provides direct functional insights into its role in pathogenesis. The genome-wide analysis revealed a diverse family with features indicative of effector potential, including secretion signals, GPI anchors, and specific regulatory motifs. Crucially, transient expression assays validated the immunosuppressive function of key members, identifying SsCFEM4 and SsCFEM9 as potent suppressors of host cell death. This experimental confirmation establishes SsCFEM9 as a functional effector candidate and uncovers a previously unannotated function for SsCFEM4. Together with the distinct phylogenetic clades and infection-induced expression patterns, our findings portray the SsCFEM family as a dynamically regulated toolkit evolved for host manipulation. This work not only provides a foundational resource but also delivers prioritized, functionally characterized candidates for future mechanistic studies, advancing the quest for targeted control strategies against this devastating pathogen.

## Figures and Tables

**Table 1:** Summary of sequence-derived physicochemical properties of the SsCFEM proteins in *S. sclerotiorum*.

Name	Gene ID	Protein ID	Amino Acid (aa)	Molecular Weight (KDa)	Theoretical pI	Instability Index	GRAVY
SsCFEM1	*APA11622.1*	sscle_08g063920	222	20.67	4.81	44.76	0.356
SsCFEM2	*APA13836.1*	sscle_11g086060	529	51.22	3.45	55.79	−0.275
SsCFEM3	*APA12577.1*	sscle_09g073470	343	38.09	8.50	40.06	0.402
SsCFEM4	*APA08632.1*	sscle_04g034020	422	46.94	8.10	36.81	0.423
SsCFEM5	*APA16345.1*	sscle_16g111150	244	22.53	4.66	39.86	0.360
SsCFEM6	*APA13624.1*	sscle_11g083940	500	55.60	8.67	38.42	0.178
SsCFEM7	*APA12107.1*	sscle_09g068770	339	31.32	3.98	42.66	0.464
SsCFEM8	*APA10258.1*	sscle_06g050280	223	21.12	4.53	42.48	0.570
SsCFEM9	*APA08792.1*	sscle_04g035620	182	17.68	4.14	37.48	0.172
SsCFEM10	*XM_001595472.1*	XP_001595522.1	119	12.25	5.10	53.35	0.397
SsCFEM11	*APA05709.1*	sscle_01g004790	715	77.03	9.38	56.94	−0.520
SsCFEM12	*APA16135.1*	sscle_16g109050	995	91.69	9.42	64.03	−0.670
SsCFEM13	*APA06944.1*	sscle_02g017140	184	17.20	4.29	36.5	0.457

The table lists thirteen identified CFEM-containing proteins (SsCFEM1 to SsCFEM13) from *S. sclerotiorum* strain 1980 UF-70. Values for amino acid length, molecular weight (MW), theoretical isoelectric point (pI), instability index, and grand average of hydropathicity (GRAVY) were calculated using the ExPASy ProtParam tool. Proteins with an instability index below 40 were predicted to be stable. A positive GRAVY value indicates overall hydrophobicity, while a negative value indicates hydrophilicity. Gene and protein identifiers are derived from the corresponding genome annotation.

**Table 2:** Bioinformatic analysis of domain architecture, subcellular localization signals, and effector potential of the SsCFEM proteins.

Name	Amino Acid (aa)	Position of CFEM Domain (aa)	Cys% in Matured Protein	TM No.	SP Cleavage	Subcellular Localization Prediction Value					GPI-Anchored	Effector
						mTP	SP	Other	Loc	RC		
SsCFEM1	222	25–90	3.90%	0	18/19	0	0.9979	0.0021	S	1	P192/P200	-
SsCFEM2	529	37–104, 189–236	3.30%	0	17/18	0	0.9936	0.0064	S	1	-	-
SsCFEM3	343	27–89	3.80%	4	-	0.0001	0.0364	0.9634	-	1	-	-
SsCFEM4	422	18–82	4.00%	5	17/18	0.0001	0.952	0.048	S	1	-	-
SsCFEM5	244	25–81	3.50%	0	18/19	0	0.9987	0.0013	S	1	P200/P216	-
SsCFEM6	500	28–92	2.70%	7	20/21	0	0.9978	0.0021	S	1		-
SsCFEM7	339	21–87	2.50%	0	18/19	0	0.9998	0.0002	S	1	P315/P308	-
SsCFEM8	223	18–86	3.90%	0	17/18	0	1	0	S	1	P200/P201	-
SsCFEM9	182	20–86	4.80%	0	16/17	0	0.9999	0.0001	S	1	P158/S162	Y
SsCFEM10	119	26–91	7.90%	0	18/19	0.0001	0.9998	0.0001	S	1	-	Y
SsCFEM11	715	20–87	1.30%	1	-	0	0.4606	0.5393	-	5	-	-
SsCFEM12	995	95–159	1.40%	1	-	0	0.002	0.998	-	1	-	-
SsCFEM13	184	22–85	6.10%	0	19/20	0	0.9996	0.0004	S	1	P160/P155	Y

The table presents key structural features and in silico predictions for the subcellular localization and functional role of the 13 SsCFEM proteins. The position of the CFEM domain(s) within the amino acid sequence is indicated. Predictions were performed using the following tools: transmembrane helices (TM) by TMHMM 2.0; signal peptides (SPs) and their cleavage sites by SignalP-4.1; mitochondrial targeting peptides (mTPs) by TargetP 2.0; GPI-anchor modification sites by the big-PI Fungal Predictor; and effector likelihood by EffectorP 2.0. Subcellular localization (Loc) was predicted based on the identified targeting signals: mTP (mitochondrial targeting peptide), SP (secretory pathway signal), and Other (any other localization). The prediction results in Loc: “S” indicates a secretory pathway, and “-” indicates that no specific localization. The reliability class (RC) indicates prediction confidence, with a value of 1 being the most reliable. For GPI-anchor and effector predictions, a specific amino acid position denotes a predicted modification site; “Y” indicates a positive prediction, and a dash (“-”) indicates no prediction was made. Proteins lacking a predicted signal peptide and transmembrane domain(s) were classified as candidate secreted proteins.

## Data Availability

Data supporting the findings of this study are available within the article or its Supplementary Materials.

## Supplementary Materials

The following supporting information can be downloaded at: https://www.mdpi.com/article/10.3390/plants15060957/s1, Table S1: Amino acid sequence of the SsCFEM proteins; Figure S1: CMXR staining for mitochondrial localization in hyphae. The mitochondria were stained with 1 mM MitoTracker™ Red CMXR (Chloromethyl-X-rosamine, Invitrogen) for 45 min at room temperature in the dark and visualized using confocal microscopy. Scale bars = 10 µm; Table S2: Sequences of the CFEM domain in the SsCFEM proteins; Table S3: Amino acid sequences of CFEM domain-containing proteins from *M. oryzae*, *B. cinerea*, *F. graminearum*, *C. albicans*, and two proteins from *U. maydis* (PAND_027d6170 and UMAG_02820) were subjected to phylogenetic analysis; Table S4: Coding sequences (CDSs) of *SsCFEMs*; Table S5: Genomic sequence of *SsCFEMs*; Table S6: Primers used in this study.
