# Supplementary material for: Genome-Wide Identification of CFEM Proteins in Sclerotinia sclerotiorum Reveals Effector Candidates with Cell Death Suppression Activity
Source: Plants (Basel). 2026 Mar 20;15(6):957. doi: 10.3390/plants15060957 (PMC13030711; doi:10.3390/plants15060957)
Supplement: Supplementary file 1 [file plants-15-00957-s001.zip › Supplementry/Supplementry Figure S1.docx]

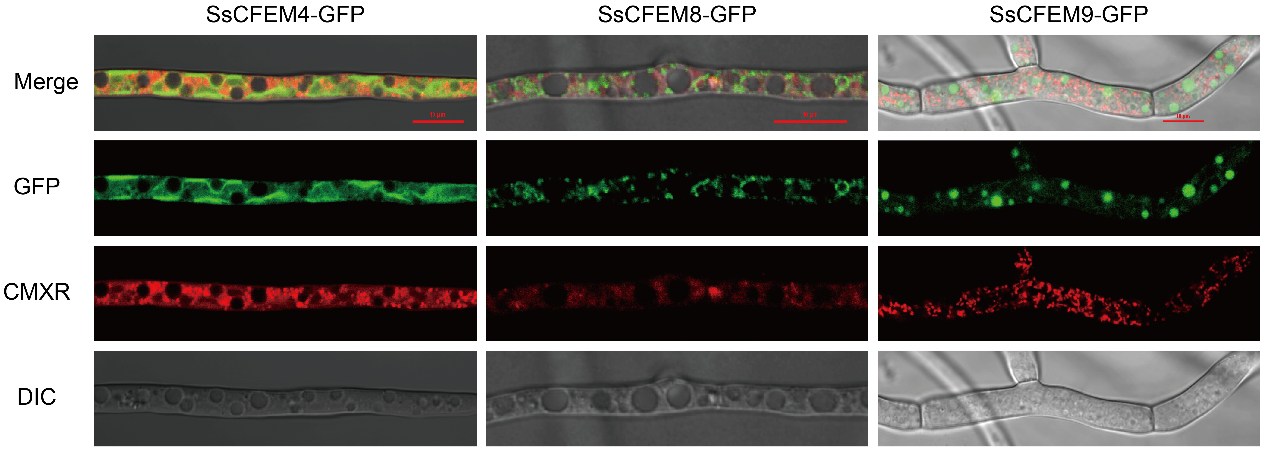
Figure S1: CMXR staining for mitochondrial localization in hyphae. The mitochondria were stained with 1 mM MitoTracker™ Red CMXR (Chloromethyl-X-rosamine, Invitrogen ‌) for 45 min at room temperature in the dark and was visualized using confocal microscopy. Scale bars = 10 µm
